# *Chlamydophila spp. *infection in horses with recurrent airway obstruction: similarities to human chronic obstructive disease

**DOI:** 10.1186/1465-9921-9-14

**Published:** 2008-01-29

**Authors:** Dirk Theegarten, Konrad Sachse, Britta Mentrup, Kerstin Fey, Helmut Hotzel, Olaf Anhenn

**Affiliations:** 1 Institute of Pathology and Neuropathology, University Duisburg-Essen Medical School, Hufelandstr. 55, D-45122 Essen, Germany; 2 Institute of Bacterial Infections and Zoonoses, Friedrich-Loeffler-Institute, Naumburger Str. 96a, D-07743 Jena, Germany; 3 Clinic for Horses, Internal Medicine, Justus-Liebig-University, Frankfurter Str. 126, D-35392 Gießen, Germany; 4 Institute of Molecular Pathogenesis, Friedrich-Loeffler-Institute, Naumburger Str. 96a, D-07743 Jena, Germany; 5 Clinic for Internal Medicine, General Hospital Hagen, Grünstr. 35, D-58095 Hagen, Germany; 6 Department of Pneumology, Ruhrlandklinik, University Duisburg-Essen Medical School, Tüschener Weg 40, D-45239 Essen, Germany

## Abstract

**Background:**

Recurrent airway obstruction (RAO) in horses is a naturally occurring dust-induced disease mainly characterized by bronchiolitis which shows histological and pathophysiological similarities to human chronic obstructive pulmonary disease (COPD). In human COPD previous investigations indicated an association with *Chlamydophila psittaci *infection. The present study was designed (1) to clarify a possible role of this infectious agent in RAO and (2) to investigate the suitability of this equine disorder as a model for human COPD.

**Methods:**

Clinico-pathological parameters of a total of 45 horses (25 horses with clinical signs of RAO and 20 clinically healthy controls) were compared to histological findings in lung tissue samples and infection by *Chlamydiaceae *using light microscopy, immunohistochemistry, and PCR.

**Results:**

Horses with clinical signs of RAO vs. controls revealed more inflammatory changes in histology (p = 0.01), and a higher detection rate of *Chlamydia psittaci *antigens in all cells (p < 0.001) and bronchiolar epithelial cells alone (p < 0.001) by immunohistochemistry. The abundance of chlamydial inclusions increased with the severity of disease. PCR was positive in 60% of horses with RAO vs. 45% of the controls (p = 0.316). *Omp*A sequencing identified *Chlamydophila psittaci *(n = 9) and *Chlamydophila abortus *(n = 13) in both groups with no significant differences. Within the group of clinically healthy horses subgroups with no changes (n = 15) and slight inflammation of the small airways (n = 5) were identified. Also in the group of animals with RAO subgroups with slight (n = 16) and severe (n = 9) bronchiolitis could be formed. These four subgroups can be separated in parts by the number of cells positive for *Chlamydia psittaci *antigens.

**Conclusion:**

*Chlamydophila psittaci *or *abortus *were present in the lung of both clinically healthy horses and those with RAO. Immunohistochemistry revealed acute chlamydial infections with inflammation in RAO horses, whereas in clinically healthy animals mostly persistent chlamydial infection and no inflammatory reactions were seen. Stable dust as the known fundamental abiotic factor in RAO is comparable to smoking in human disease. These results show that RAO can be used as a model for human COPD.

## Background

Recurrent Airway Obstruction (RAO) in horses, formerly called equine chronic obstructive pulmonary disease (COPD), is a very common illness [1]. Pathology is characterized by bronchiolitis [2,3], which is similar to findings in human COPD and advanced human pulmonary emphysema [4]. Organic dust exposure and endotoxin are relevant factors for chronic impairment of lung function in horses and are used in experimental animal studies to cause exacerbations [5,6].

Our own group detected *Chlamydophila psittaci (CPP) *infection by PCR in 38% of human patients with COPD [7,8], who were suffering from advanced pulmonary emphysema and underwent lung volume reduction surgery. Chlamydial antigens were shown in alveolar parenchyma and bronchioles. *CPP *and also *Chlamydophila abortus (CPA) *was detected in sputum samples from patients with exacerbations of COPD as well [9]. Up to now, *Chlamydophila *spp. have been rarely detected in horses with acute respiratory infections [10,11]. Mair and Wills [12] found culturable chlamydiae in 5% of equine nasal and conjunctival swabs in a prevalence study, but no association between isolation and clinical diseases was seen. However, these low detection rates in the pre-PCR era may have been due to the known difficulties of culturing these obligate intracellular bacteria. Serologically antibody titres ≥ 32 against *Chlamydophila pneumoniae (CPPN) *were detected in 26.5% of Italian light horses. Some sera with high titres reacted weakly with *CPP *as well [13]. But within aborted equine fetuses *CPP *has been found in high prevalence [14,15]

In the present study lung tissue samples from horses with RAO as well as a group of clinically healthy horses were examined by immunohistochemistry, immunofluorescence and PCR to assess the extent of chlamydial infection and its association with RAO. PCR positive samples were additionally evaluated by DNA sequencing to define exactly the chlamydial species involved.

## Methods

### Samples

Out of 948 horses, which were slaughtered or euthanized in the period between November 2002 and October 2004 in three western districts of Westphalia, 26 horses were identified as possibly suffering from RAO by questioning veterinarians and/or horse owners about reasons for slaughter or euthanasia. Slaughter was done according to federal law in authorized slaughterhouses and under control of a veterinarian. Euthanasia was practised by veterinarians following internationally recognized guidelines. If the horses had shown respiratory disease history, symptoms and therapy as well as conditions of housing were evaluated in detail by using a standardized questionnaire, which is available as online addendum. Possible RAO and control horses were clinically examined before slaughter or euthanasia. The horses had to meet the following criteria [Additional file 1]: (1) chronic cough for at least 3 months duration; (2) exercise intolerance; (3) worsening of the symptoms due to dust; (4) obvious biphasic expiratory dyspnoea with hypertrophy of Mm. recti abdomini, inflated nostrils and nasal discharge; (5) breathing frequency > 20/min; and (6) pathologic findings in lung auscultation. As respiratory healthy controls served 20 horses which completely lacked these clinical criteria. Immediately after death, tissue was taken from 8 different lung regions of each horse and preserved for the following examinations.

### Light Microscopic Histology

Formalin-fixed lung tissue was embedded in paraffin wax (Tissuewax™; Medite GmbH, Burgdorf, Germany), slices of 3–7 μm thickness were cut using a rotatory microtome (Microm GmbH, Walldorf, Germany), and stained with haematoxylin and eosin. Slices from the 8 tissue samples of each horse were evaluated for histological changes using a semi-quantitative score (0–4) in a blinded study. No signs of bronchiolitis were scored with 0 points. Minimal infiltration of the bronchioli with lymphocytes was scored with 1, slight inflammation with 2, moderate bronchiolitis with some neutrophils with 3, and severe changes with intraluminal aggregates of neutrophils with 4 points. Results from all 8 regions of each horse were added. Therefore a minimal score of 0 and a maximal score of 32 was possible.

One horse with RAO-like respiratory symptoms and history revealed parasitic lung disease after histological investigation and was excluded therefore.

According to anamnesis, clinical findings and light microscopy, the remaining 45 horses were classified into four subgroups:

I. Clinically healthy horses without histological changes of RAO (n = 15),

II. Clinically healthy horses with a low inflammation score of 4–6 (n = 5),

III. Horses with clinical signs of RAO, but without or with only slight histological changes and a score of 0–5 (n = 16), and

IV. Horses with symptoms and histological changes of RAO with a score > 10 (n = 9).

### Immunohistochemistry and Immunofluorescence

For immunohistochemistry, dewaxed slices were stained with a mouse anti-*CP *primary antibody (Biotrend, Germany; clone 73/0200, dilution 1:60) for 2 hours at room temperature, followed by a peroxidase-based detection system (Histostain^®^-Bulk-Kit, Zymed Laboratories Inc., San Francisco, USA) according to the instructions of the manufacturer). Three high-power fields showing bronchioli were selected for counting all detectable vs. all *CP*-positive stained bronchiolar epithelial cells (BEC), type II pneumocytes (PII) and macrophages (MP) separately, using a Zeiss Axiophot microscope (Carl Zeiss, Oberkochen, Germany) with a 40-fold objective magnification and a digital imaging system (Diskus Software, Koenigswinter, Germany). For immunofluorescence, triple staining on dewaxed sections was done with a primary mouse monoclonal IgG_2b_-antibody against *CP *(Biotrend, Germany; clone 73/0200, dilution 1:100) and a primary mouse monoclonal IgG_1_-antibody mix against pan-cytokeratin (BioCarta, USA; clones AE1, AE3, 5D3, dilution 1:50), as well as 4',6-diamidino-2-phenylindol-hydrochloride (DAPI, Sigma, Germany, working concentration 0.1 μg/ml). Alexa Fluor 488-conjugates of goat anti-mouse-IgG_1 _(dilution 1:400), and Alexa Fluor 594-conjugates of goat anti-mouse-IgG_2b _(dilution 1:400) were used as secondary antibodies (Molecular Probes Europe, Leiden, The Netherlands). Results were documented with a CCD-camera (Kappa, Gleichen, Germany) attached to an Olympus IX-70 microscope (Olympus Europe, Hamburg, Germany) with 40-fold objective magnification.

### Polymerase Chain Reaction (PCR)

Lung tissue was collected immediately after slaughtering/euthanizing of the horses and stored at -80°C. DNA was isolated from lung tissue using the High Pure PCR Template Preparation Kit (Roche Diagnostics, Mannheim, Germany) according to the instructions of the manufacturer. 5 μl of the DNA extract were used as template in PCR. Samples were tested for *CPP/CPA *and *CPPN *by a modified version of the nested PCR procedure described by Kaltenboeck et al. [16] which targets the *omp*A gene. The first step was genus-specific amplification using primers 191CHOMP (5'-GCI YTI TGG GAR TGY GGI TGY GCI AC-3') and CHOMP371 (5'-TTA GAA ICK GAA TTG IGC RTT IAY GTG IGC IGC-3'). For the second amplification, we used 1 μl of the genus-specific product and primer combination 218PSITT (5'-GTA ATT TCI AGC CCA GCA CAA TTY GTG-3')/CHOMP336s (5'-CCR CAA GMT TTT CTR GAY TTC AWY TTG TTR AT-3') for *CP*, or 201CHOMP (5'-GGI GCW GMI TTC CAA TAY GCI CAR TC-3')/PNEUM268 (5'-GTA CTC CAA TGT ATG GCA CTA AAG A-3'), for *CPPN*, respectively. The sizes of specific amplicons are: 576–597 bp (genus-specific product), and 389–404 bp for *CP*, or 244 bp for *CPPN *after nested PCR. A detailed protocol of the procedure was previously published by Sachse and Hotzel [17] and is given here:

#### A. Genus-Specific Detection of Chlamydiae

Prepare a master mix of reagents for all amplification reactions of the series. It should contain the following ingredients per 50-μl reaction:

1 μl dNTP mix (2 mM each), 1 μl primer 191CHOMP (20 pmol/μl), 1 μl primer CHOMP371 (20 pmol/μl), 5 μl reaction buffer (10×), 0.2 μl *Taq *DNA polymerase (5 U/μl), 40.8 μl H_2_O. Add template to each reaction vessel: 1 μl of DNA extract from infected tissue or 5 μl of extract from swab samples. Include amplification controls: DNA of a chlamydial reference strain (positive control) and water (negative control 1) instead of sample extract. Run PCR according to the following temperature-time profile: Initial denaturation at 95°C for 30 s, 35 cycles of denaturation (95°C for 30 s), primer annealing (50°C for 30 s) and primer extension (72°C for 30 s). Correct amplification leads to the formation of a 576–597-bp product specific for the genus *Chlamydia *(according to the new taxonomy: *Chlamydia *and *Chlamydophila*).

#### B. Species-Specific Detection of Chlamydiae

Prepare a master mix of reagents for all amplification reactions of the series. It should contain the following ingredients per 50-μl reaction:

1 μl dNTP mix (2 mM each), 1 μl forward primer 201CHOMP + 1 μl reverse primer TRACH269 or PNEUM268 (20 pmol/μl each) OR 1 μl forward primer 204PECOR or 218PSITT + 1 μl reverse primer CHOMP336s (20 pmol/μl each), 5 μl reaction buffer (10×), 0.2 μl *Taq *DNA polymerase (5 U/μl), 40.8 μl H_2_O. Add 1 μl of the product from genus-specific PCR as template to each reaction vessel.

Subject the products of positive control and negative control 1 (1 μl of each) from the previous amplification to the second round of nested PCR. Additionally include a fresh reagent control (negative control 2). Run PCR according to the following temperature-time profile: Initial denaturation at 95°C for 30 s, 20 cycles of denaturation (95°C for 30 s), primer annealing (60°C for 30 s) and primer extension (72°C for 30 s). The correct sizes of species-specific amplicons are 250 bp for *C. trachomatis*, 244 bp for *C. pneumoniae*, 389–404 bp for *C. psittaci*, and 426–441 bp for *C. pecorum*.

### DNA sequencing

For species identification, products of the first PCR round were amplified using primers 201CHOMP and CHOMP336s. Specific bands from PCR products were cut out of the agarose gel (1%) and DNA was extracted using the QIAquick Gel Extraction Kit (QIAGEN, Hilden, Germany). Prior to processing on the ABI PRISM 310 Genetic Analyzer (Applied Biosystems), these extracts were subjected to cycle sequencing using the same primers as above and the BigDye™ Terminator Cycle Sequencing Ready Reaction Kit (Applied Biosystems, Darmstadt, Germany). The average length of the sequences determined was 400 nucleotides. The species identity was established through BLAST search [18]. Sequence alignments and analysis were conducted using the Vector NTI Suite 8.0 software package (Informax Inc., Oxford, UK).

### Statistical analysis

Statistical analysis was done using SPSS, version 12 (SPSS Inc., Chicago, USA). The data were statistically evaluated using Mann-Whitney-U-test, Pearson Chi Square test and Spearman-Rho correlation (2-sided). A test value below 0.05 was considered to be statistically significant.

## Results

### Clinical data

As a result of the standardized questionnaire and histology 45 out of a total of 948 horses were recruited for the present study. 43 animals were slaughtered and 2 euthanized. The median age was 16.11 years (span: 9–25), among these were 23 mares, 20 geldings and 2 stallions. Concerning the respiratory system, 20 animals were considered clinically as healthy, whereas 25 showed clinical symptoms of RAO (Table 1). Lameness was the main cause to kill the horses (40% in both groups). In horses with RAO, this disease was the reason for killing the animal in 36%; a respective therapy was applied in 56%. Therapeutic regimens differed widely, thus not allowing further statistically evaluation. The majority of horses both in the clinically healthy and the RAO groups were housed in stables with straw bedding (healthy controls: 75%, RAO: 68%). Outdoor housing was reported in 20% of the controls and none of the histologically approved RAO cases. Stable housing with wood shavings or linen straw was reported in 32% of cases with RAO and 5% of controls, respectively.

**Table 1:** Findings in clinically healthy versus clinically sick horses

	**n**	**Histological score: median (span)**	**IHC (MP): median (span)**	**IHC (PII): median (span)**	**IHC (BE): median (span)**	**IHC (percentage): median (span)**	**PCR: positive cases (percentage)**
**Clinically healthy **(Subgroups I + II)	20	0 (0–6)	0 (0–1)	0 (0–4)	17 (0–119)	1.108 (0–8.92)	9 (45)
**Clinically sick **(Subgroups III + IV)	25	3 (0–29)	0 (0–4)	0 (0–63)	113 (4–535)	7.304 (0.20–37.1)	15 (60)
Mann-Whitney-U *Pearson Chi Square		p = 0.010	p = 0.079	p = 0.127	p < 0.001	p < 0.001	*p = 0.316

Immunohistochemistry (IHC): Number of CP antigen-positive cells in 3 high power fields

MP: macrophages, PII: type II pneumocytes, BE: bronchiolar epithelial cells

IHC (percentage): Number of all CP antigen-positive cells in 3 high power fields in relation to all cells

### Light microscopy, immunohistochemistry and Immunofluorescence

Comparing RAO and healthy horses (Table 1) a significantly different histological score (p = 0.010), number of *Chlamydia psittaci (CP) *antigen-positive bronchiolar epithelial cells (p < 0.001), and percentage of all *CP *antigen-positive cells (p < 0.001) were found.

In subgroups II and III the distribution of inclusions and antigens was typical for persistent chlamydial infections in IHC: inclusion bodies were only sparsely seen, but antigens were much more frequently found. This was also true for subgroup 1, but on a much lower level.

Animals which were killed specifically because of RAO (n = 9) were significantly different in the median number of *CP *antigen-positive bronchiolar epithelial cells (83 vs. 42, p = 0.037) compared to horses which were put down due to other reasons (n = 36).

Results of clinical data and histological evaluation of the 45 horses led to the classification shown in Table 2. The four subgroups were significantly different from each other in their light microscopy histological score (Mann-Whitney U-test, p < 0.05). Age was not significantly correlated to the histological score (Spearman-Rho, p = 0.425).

**Table 2:** Comparison of results after subgrouping horses due to clinical and light microscopic findings

	**n**	**Histological score: median (span)**	**IHC (MP): median (span)**	**IHC (PII): median (span)**	**IHC (BE): median (span)**	**IHC (percentage): median (span)**	**PCR: positive cases (percentage)**
**Subgroup I**	15	0 (0–2)	0 (0–1)	0 (–-4)	6 (–-63)	0.359 (0–4,79)	5 (33.3)
**Subgroup II**	5	5 (4–6)	0 (0–1)	0 (0–2)	76 (11–119)	4.853 (0.88–8.92)	4 (80)
**Subgroup III**	16	1 (0–5)	0 (0–1)	0 (0–2)	81.5 (4–474)	6.065 (0.20–25.4)	13 (81.2)
**Subgroup IV**	9	22 (12–29)	1 (0–4)	22 (0–63)	246 (20–535)	21.024 (1.37–37.1)	2 (22.2)
Mann-Whitney-U *exact Fisher test		I vs. II: p < 0.001 I vs. III: p = 0.033 I vs. IV: p < 0.001 II vs. III: p = 0.003 II vs. IV: p = 0.001 III vs. IV: p < 0.001	I vs. II: p = n. s. I vs. III: p = n. s. I vs. IV: p = 0.041 II vs. III: p = n. s. II vs. IV: p = n. s. III vs. IV: p = 0.037	I vs. II: p = n. s. I vs. III: p = n. s. I vs. IV: p < 0.001 II vs. III: p = n. s. II vs. IV: p = 0.007 III vs. IV: p < 0.001	I vs. II: p = 0.033 I vs. III: p < 0.001 I vs. IV: p < 0.001 II vs. III: p = n. s. II vs. IV: p = 0.042 III vs. IV: p = 0.023	I vs. II: p = 0.015 I vs. III: p < 0.001 I vs. IV: p < 0.001 II vs. III: p = n. s. II vs. IV: p = 0.042 III vs. IV: p = 0.020	I vs. II: p = n. s.* I vs. III: p = 0.019* I vs. IV: p = n. s.* II vs. III: p = n. s.* II vs. IV: p = n. s.* III vs. IV: p = 0.009*

Subgroup I: Clinically healthy horses without histological changes of RAO

Subgroup II: Clinically healthy horses with a low inflammation score of 4–6

Subgroup III: Horses with clinical signs of RAO, but without or only slight histological changes and a score of 0–5

Subgroup IV: Horses with symptoms and histological changes of RAO with a score > 10

IHC: Number of CP antigen-positive cells in 3 high power fields

MP: macrophages, PII: type II pneumocytes, BE: bronchiolar epithelial cells

IHC (percentage): number of all CP antigen-positive cells in 3 high power fields in relation to all cells

**Figure 1:**
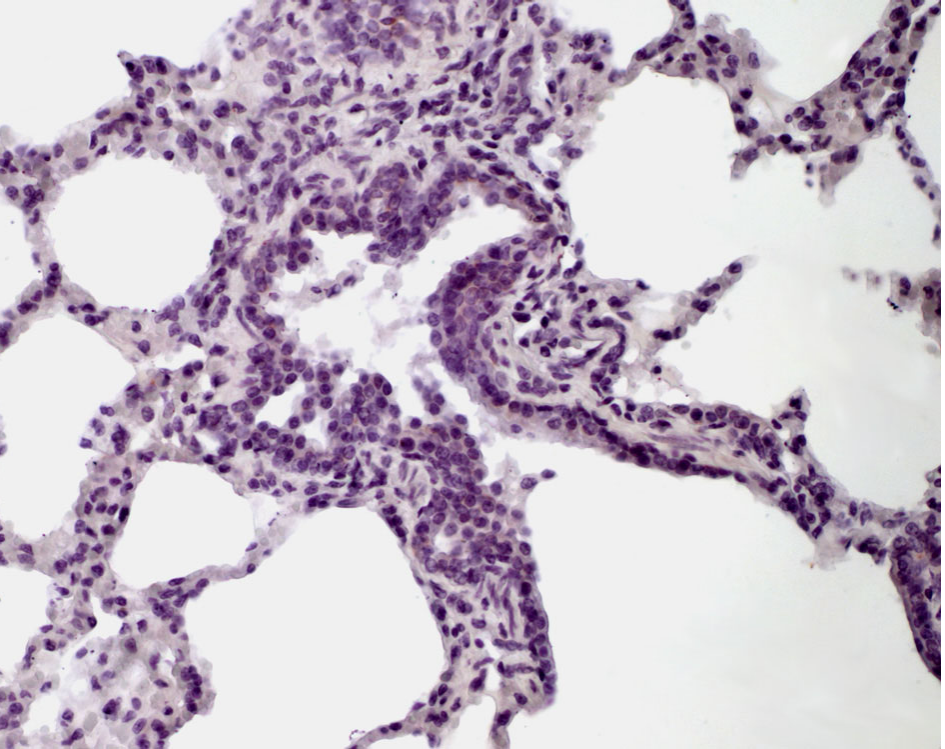
Immunohistochemistry of equine lung tissue in healthy horses. Animal showing no bronchiolitis (original magnification 10×).

**Figure 2:**
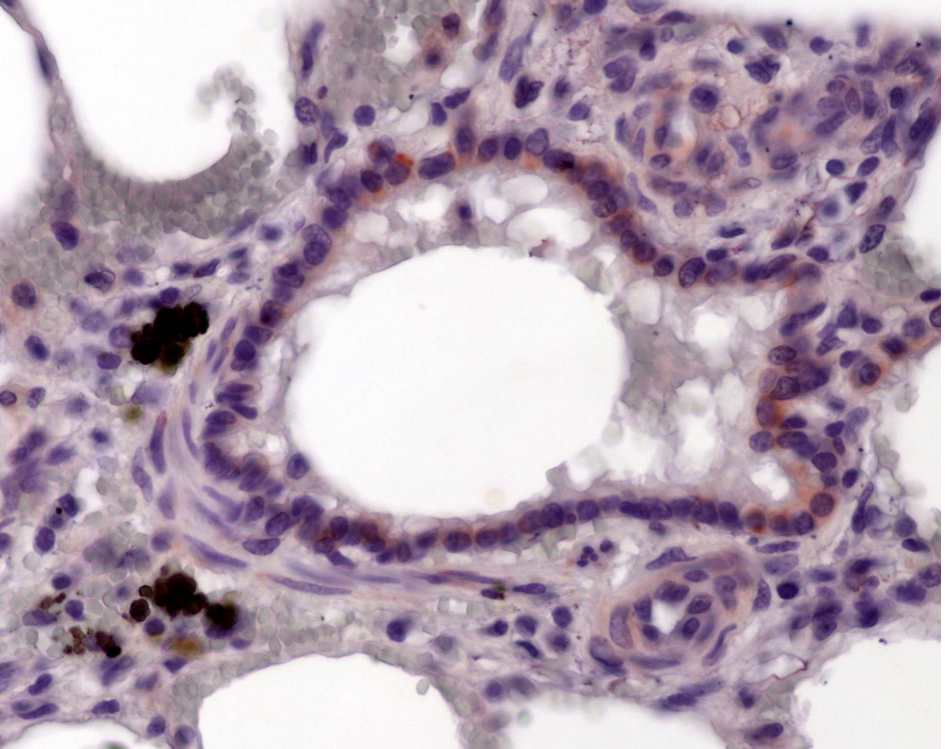
Immunohistochemistry of equine lung tissue in healthy horses. Only occasionally some epithelial cells positive for *Chlamydia psittaci *antigens are detectable (brown staining, original magnification 40×).

**Figure 3:**
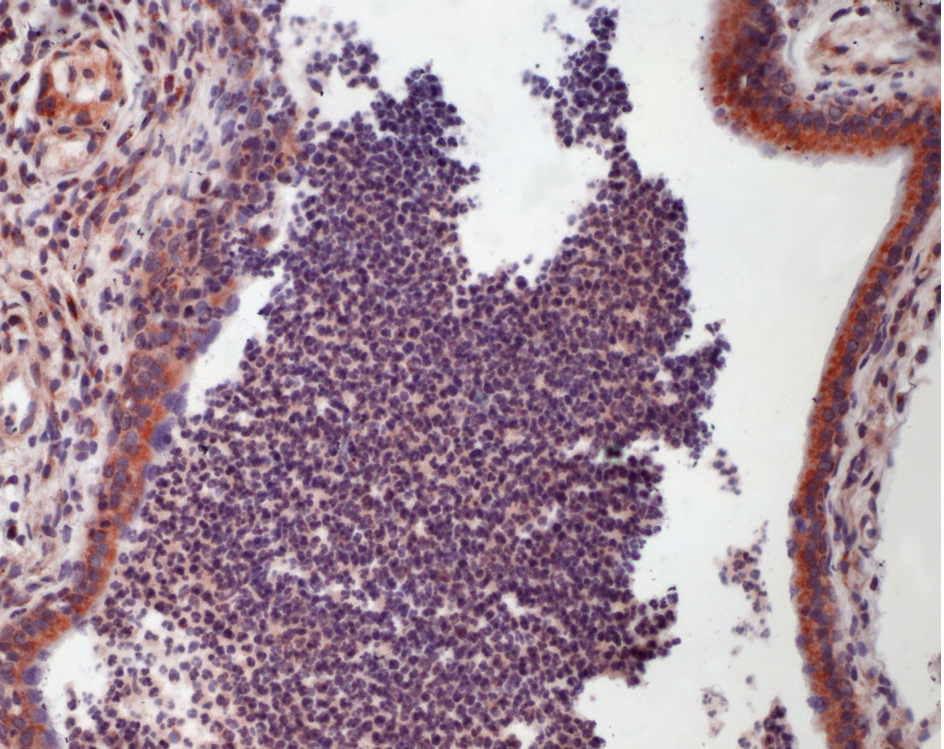
Immunohistochemistry of equine lung tissue in RAO. In RAO, animals with severe bronchiolitis (note the striking intraluminal accumulation of neutrophils and macrophages) most of the bronchiolar epithelial cells carry *CP *antigens (brown staining, original magnification 20×).

**Figure 4:**
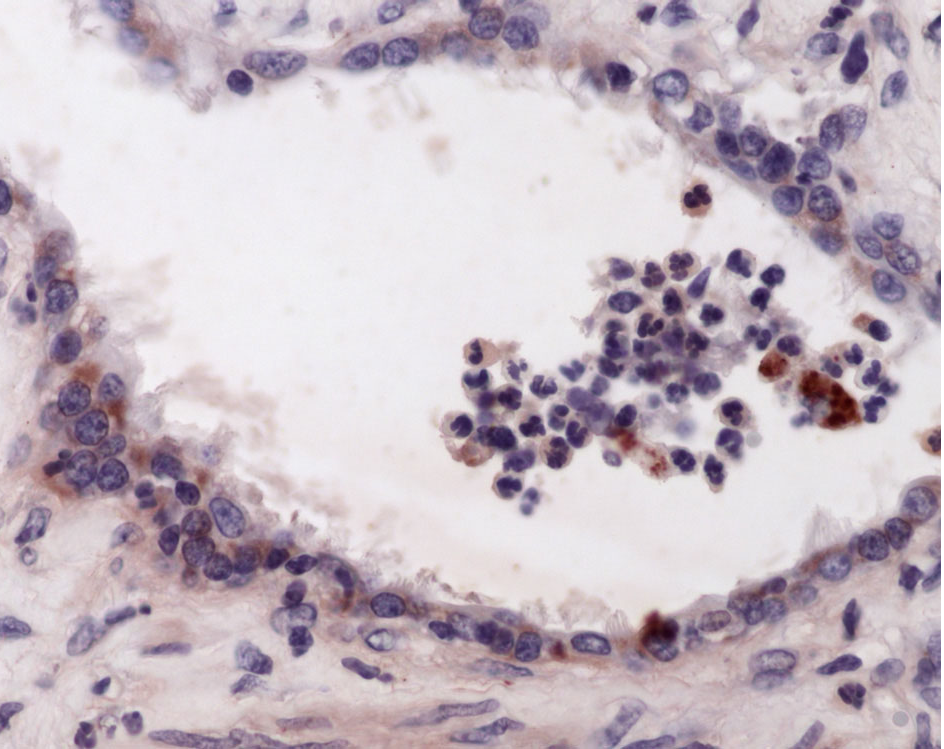
Immunohistochemistry of equine lung tissue in RAO. Also in the respiratory bronchioli inflammation is found, chlamydial antigens (brown staining) are detectable in epithelial cells and macrophages (original magnification 40×).

**Figure 5:**
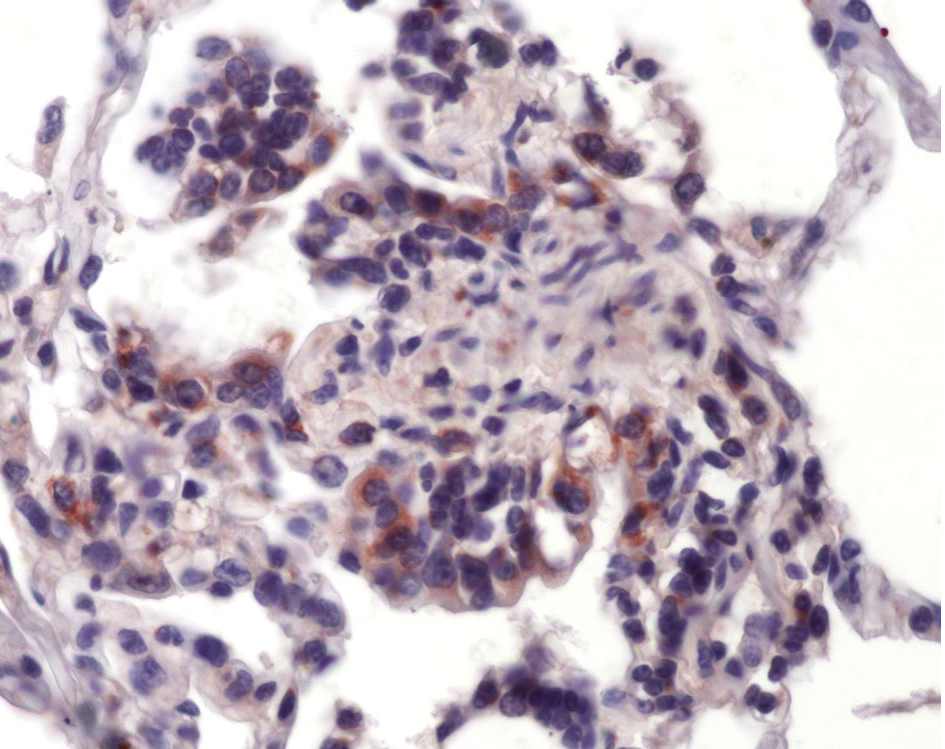
Immunohistochemistry of equine lung tissue in RAO. Proliferating type II pneumocytes show chlamydial antigens as well (brown staining, original magnification 40×).

The clinically healthy horses in subgroup I showed no (Figure 1) and in subgroup II only slight bronchiolitis. *CP *antigens were shown in bronchiolar epithelial cells (Figures 2, 3 and 4) as well as occasionally in type 2 pneumocytes and macrophages (Figures 4 and 5). Type II pneumocytes were positive in 12 cases (3 in subgroup I, 1 in subgroup III, 8 in subgroup IV), and in 6 cases positive staining was also seen in macrophages (1 in subgroup I, 5 in subgroup IV). Data and significant differences are shown in detail in Table 2. With increased severity of disease there was a significant rise in antigen-positive cells. Age was not significantly correlated with immunohistological parameters (Spearman Rho, p > 0.05). Positive and significant Spearman-Rho correlations were found between all of the following parameters: light microscopy score, the number of *CP *antigen-positive macrophages, type II pneumocytes and bronchiolar epithelial cells. However, the highest correlation coefficient (r = 0.612) was found between the number of *CP *antigen-positive type II pneumocytes and the number of *CP *antigen-positive macrophages, the lowest correlation coefficient found was r = 0.41 between the number of *CP *antigen-positive macrophages and the histological score (Table 3).

**Table 3:** Correlations between histological score and immunohistochemical findings

**Correlation coefficients **(Spearman-Rho, 2-sided)	**Histological score**	**IHC (MP)**	**IHC (PII)**
**IHC (MP)**	0.410 p = 0.005		
**IHC (PII)**	0.459 p = 0.002	0.612 p < 0.001	
**IHC (BE)**	0.493 p = 0.001	0.448 p = 0.002	0.495 p = 0.001

IHC: Number of CP antigen-positive cells in 3 high power fields

MP: macrophages, PII: type II pneumocytes, BE: bronchiolar epithelial cells

**Figure 6:**
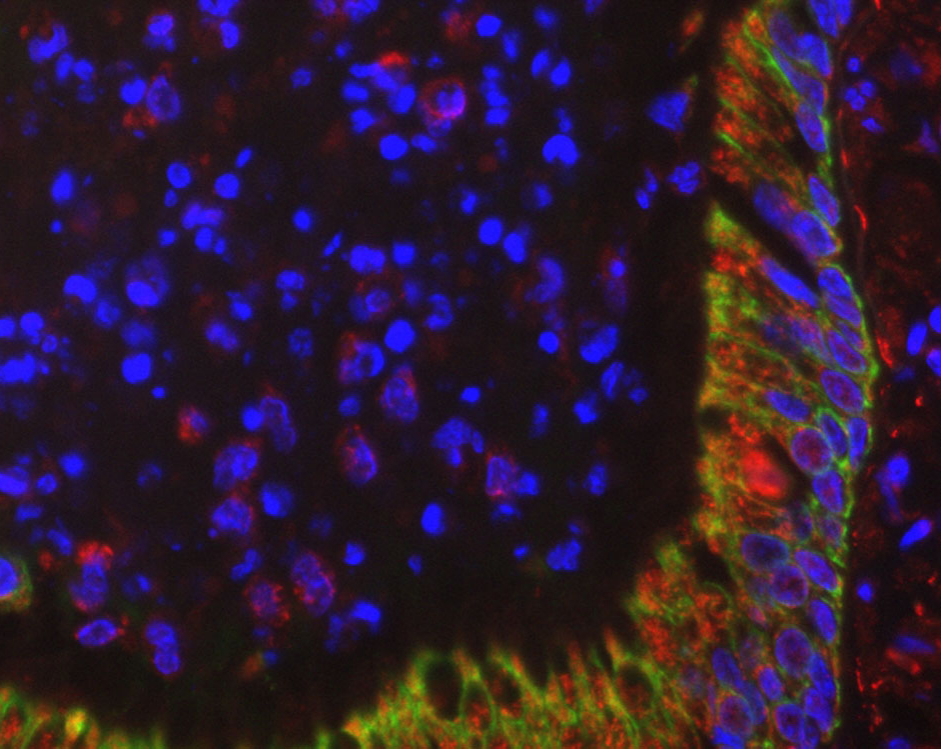
Immunofluorescence of equine lung tissue in RAO. In horses with RAO, the abundance of chlamydial inclusion bodies (red spots) in bronchiolar epithelial cells (stained green with an anti-cytokeratin pan antibody) and macrophages varies from high to low (compare Figure 7) (original magnification 40×).

**Figure 7:**
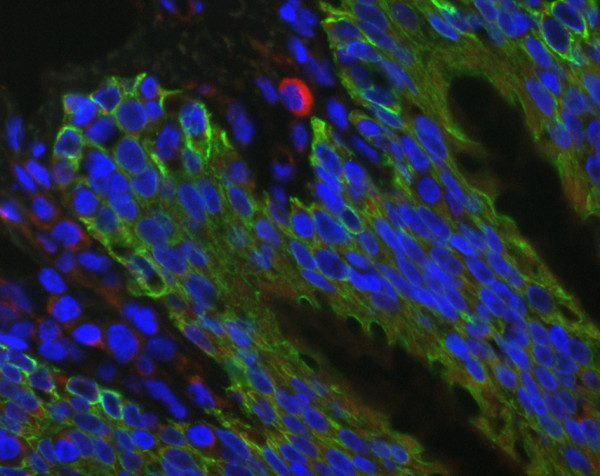
Immunofluorescence of equine lung tissue in RAO. In horses with RAO, the abundance of chlamydial inclusion bodies (red spots) in bronchiolar epithelial cells (stained green with an anti-cytokeratin pan antibody) and macrophages varies from high (compare Figure 6) to low (original magnification 40×).

To obtain further information about localization of chlamydial antigens, immunofluorescence testing was performed in three horses. Multiple typical perinuclear spots in bronchiolar epithelial cells, macrophages and granulocytes were seen in 2 horses of subgroup IV (Figures 6 and 7). In horses with RAO, the abundance of chlamydial inclusion bodies (red spots) in bronchiolar epithelial cells (stained green with an anti-cytokeratin pan antibody) and macrophages varies from high (Figure 6) to low (Figure 7).

Housing with straw contact vs. other bedding or RAO with therapy vs. without therapy and all combinations of the parameters histological score, results of immunohistochemistry or PCR, lacked any correlations (Mann-Whitney U-test, p > 0.05).

### Polymerase Chain Reaction (PCR) and DNA sequencing

No significant differences were seen between clinically healthy and sick horses (Table 1). PCR revealed *CP *DNA in 5 of 15 (= 33.3%) horses in subgroup I, 4 of 5 (= 80%) in subgroup II, 13 of 16 (= 81.2%) in subgroup III and 2 of 9 (= 22.2%) in subgroup IV. Significant differences in PCR positivity are shown between subgroups I and III as well as between subgroups III and IV (Table 2).

To confirm the identity of the chlamydial species, DNA from 22 of the 24 positive samples was sequenced in the *omp*A gene region (approximately 400 bp). A BLAST search of these sequences revealed close to 100% homology to the species *CPP *in 9 and *CPA *in 13 cases. No significant differences were found between the four subgroups (Pearson Chi Square test, p = 0.309).

## Discussion

One principal characteristic of RAO is the excessive production of mucus containing high amounts of neutrophils [19]. In human COPD, mucus hypersecretion is a key event as well, but neutrophils are only dominant in exacerbations [20]. These exacerbations are mainly associated with viral and/or bacterial infections [21]. In equine RAO, high levels of collagenolytic activity [22] and matrix metalloproteinases (MMPs) 8, 9 and 13 [22-25] were found to be useful markers for ongoing disease. The release of matrix metalloproteinases (MMP-9) is shown to be stimulated by chlamydial heat shock protein 60 [26]. Release of MMPs is also crucial for tissue destruction by macrophages in human emphysema [27] and increased activities of MMP-8 and MMP-9 are found in induced sputum of humans with COPD [28]. Elevated IL-8 and tumor necrosis factor-alpha production are seen in horses with RAO exacerbation [19,29] and in humans with COPD [30]. The proinflammatory granulocyte-attracting chemokine IL-8 is produced by epithelial cells in the course of infection with *CPP *[31]. Decreased phagocytic activity of alveolar macrophages (MP) is found in RAO after 24 h stimulation with LPS or PMA and ionomycin [19]. In current non-smokers with COPD MP show reduced capacity to ingest apoptotic airway epithelial cells as well [32].

Both RAO and COPD have to be regarded as multicausal diseases with abiotic and biotic factors. The main etiologically relevant factor in both RAO and COPD is an external event (abiotic factor), i.e. environmental dust exposure in horses and cigarette smoking in human beings. Optimal housing can certainly prevent equine RAO in many cases as well as avoidance of smoking will prevent COPD. Some authors even regard human COPD as a dust-induced disease and emphasize the role of kaolinite [33]. Genetic (biotic) factors are relevant in RAO [34] and COPD as well [35].

According to the findings of the present study the predominant location for chlamydial antigens in horse lungs are the bronchial epithelial cells, followed to a much lesser extent by type II pneumocytes and macrophages. For bronchiolar epithelial cells as well as for the total percentage of *CP *antigen-positive cells, there is a highly significant higher antigen load in RAO horses in comparison to healthy controls. Since the first step in the inflammation cascade is caused by inhalation of dust particles, the bronchiolar epithelium represents most likely the "key structure" in establishing and maintaining the disease. Concurrent infection of these cells with chlamydial organisms might lead to chronicity and persistence of the exaggerated inflammatory and tissue destructing response to dust particles.

Distribution of *CP *antigens in the lungs of clinically healthy horses (subgroups I and II) was typical for persisting infection and in those with RAO and histological severe disease (subgroup IV) for acute chlamydial infection. The subgroup with slight inflammation and higher *CP *antigen load within the group of healthy horses could be an indicator of preclinical disease. The same questions come around according to the subgroups within RAO animals. Although no significant morphological differences were seen between healthy horses with slight inflammation (subgroup II) and those with RAO and low histological score (subgroup III) there are severe clinical differences, which can be interpretated only functionally, probably on a genetic background.

PCR positivity shows significant differences between subgroups I and III as well as between subgroups III and IV. This means that there is a significant difference also by PCR between healthy horses and horses that already present with clinical signs of RAO, but show no or only slight signs of inflammation and also between ill horses with only slight inflammation compared with those with marked inflammation. Furthermore, it is known that farmers more often suffer from respiratory symptoms than the average population [36]. Dust exposure and higher endotoxin concentrations in stables are supposed to be the main factors [37]. The possible importance of *Chlamydiaceae *as a source of human infections in animal housings requires further evaluation.

## Conclusion

For the first time persistence of *CPP *and *CPA *in the lungs of clinically healthy horses and acute (possibly reactivated) chlamydial infections in those with obvious disease is demonstrated. Respiratory chlamydial infection probably becomes clinically relevant only in animals affected by additional pathogenic factors. In this context, inflammation seems to be associated with the activation of chlamydiae. Furthermore, the high prevalence of these chlamydial agents in horse lungs deserves further attention as a reservoir, especially in the light of recent studies, showing an association of *CPP *with cases of equine abortion [14,15]. The possible risk to acquire *CPP *or *CPA *infections for the staff in animal housings has to be evaluated.

Corresponding pathophysiological aspects (target cell types, cytokine/chemokine release, tissue degrading enzymes like MMPs) between chlamydial infections on the one hand and RAO/COPD on the other hand are shown. The precise role of chlamydial organisms in the pathogenesis of RAO/COPD needs further investigations.

Because of the pathophysiological similarities between both diseases and the detection of *Chlamydiaceae*, RAO could be a model for human COPD and should be studied under these aspects.

## Competing interests

The author(s) declare that they have no competing interests.

## Authors' contributions

Dirk Theegarten (DT) has designed and organized this study, done light microscopy, written most of the manuscript and participitated in its statistical analysis. Konrad Sachse (KS) has done the sequence alignments and written the parts of the manuscript concerning molecular biology and veterinary aspects, and also participated in PCR analysis. Britta Mentrup (BM) has done the clinical parts, collected specimens and did the immunohistochemistry. Kerstin Fey (KF) has designed the clinical parts. Helmut Hotzel (HH) carried out PCR analysis and DNA sequencing. Olaf Anhenn (OA) participated in designing the study, established the immunohistochemistry protocols, collected the data, performed statistical analysis and reviewed the manuscript. All authors have discussed and approved the final manuscript.

## Supplementary Material

Additional file 1 RAO Questionnaire. Shows the questionnaire used to identify horses with or without RAO. Click here for file
