# Supplementary material for: Chlamydophila spp. infection in horses with recurrent airway obstruction: similarities to human chronic obstructive disease
Source: Respir Res. 2008 Jan 29;9(1):14. doi: 10.1186/1465-9921-9-14 (PMC2276488; doi:10.1186/1465-9921-9-14)
Supplement: Additional file 1 — RAO Questionnaire. Shows the questionnaire used to identify horses with or without RAO. [file 1465-9921-9-14-S1.doc]

**RAO Questionnaire** Number:

Age: Sex:

Race: Colour:

| **CRITERIA** | **Yes** | **No** |
| --- | --- | --- |
| **1. History** |  |  |
| 1.1 Persisting or recurrent coughing (over 3 months) |  |  |
| 1.2 Exercise intolerance (because of RAO) |  |  |
| 1.3 Negative effect of dust as potential antigen |  |  |
| **2. Inspection** |  |  |
| 2.1 Obvious biphasic expiratory dyspnoea |  |  |
| 2.2 Hypertrophy of Mm. recti abdomini |  |  |
| 2.3 Inflated nostrils |  |  |
| 2.4 Nasal discharge |  |  |
| **3. Examination** |  |  |
| 3.1 Breathing frequency >20/min |  |  |
| 3.2 Positive findings in auscultation |  |  |
| CONCLUSION | RAO | Non-RAO |

**Additionally:**

Therapy for RAO: yes no

if yes, which:

Housing conditions: stable straw

others:

Reason for killing: RAO other:

Slaughter Euthanasia

Special remarks:
